# Development and Validation of Machine Learning Models to Identify Emergency Department Patients at Increased Risk of New or Progressive Acute Kidney Injury

**DOI:** 10.1016/j.acepjo.2026.100397

**Published:** 2026-04-18

**Authors:** Jeremiah S. Hinson, Xihan Zhao, Michael R. Ehmann, Martin S. Copenhaver, Ilya Shpitser, Steven Menez, Chirag Parikh, Alexandra T. Strauss, Gabor Kelen, Oluwakemi Badaki-Makun, Scott Levin, Eili Klein

**Affiliations:** 1 Department of Emergency Medicine, Johns Hopkins University School of Medicine, Baltimore, Maryland, USA; 2 Malone Center for Engineering in Healthcare, Johns Hopkins University Whiting School of Engineering, Baltimore, Maryland, USA; 3 Department of Computer Science, Johns Hopkins Whiting School of Engineering, Baltimore, Maryland, USA; 4 Department of Medicine, Johns Hopkins University School of Medicine, Baltimore, Maryland, USA; 5 Department of Pediatrics, Johns Hopkins University School of Medicine, Baltimore, Maryland, USA; 6 One Health Trust, Washington, District of Columbia, USA

**Keywords:** kidney injury, machine learning, artificial intelligence, prediction, quality improvement place in informatics collection

## Abstract

**Objectives:**

Acute kidney injury (AKI) is a common and serious condition associated with prolonged hospitalization, chronic kidney disease, and increased mortality. Early prediction of AKI offers an opportunity to mitigate these adverse outcomes, yet existing models often fail to generalize to the emergency department (ED) setting, particularly for patients discharged directly to the community. We sought to develop and validate machine learning models that predict new or progressive AKI within 72 hours of ED departure, addressing challenges related to missing outcome data for discharged patients.

**Methods:**

This retrospective, multicenter study of adult patients from 5 EDs within a large health-care system included adult patients with at least 1 serum creatinine measurement during their ED visit. AKI was defined using Kidney Disease Improving Global Outcomes serum creatinine-based criteria, and prediction models relied on demographic, clinical, and laboratory data routinely collected during ED care. Extreme gradient boosting algorithms were trained using 4 approaches to handle missing outcome data: incomplete case exclusion, negative outcome assumption, multiple imputation, and inverse probability weighting. Model performance was evaluated via 10-fold cross-validation and external temporal validation using area under the receiver operating characteristic curve, precision, recall, calibration curve analyses, and measurement of diagnostic performance across a range of risk thresholds.

**Results:**

A total of 1,124,017 ED visits between 2017 and 2024 were included in the study; 5.7% (22,093) met AKI progression outcome criteria. The models demonstrated robust predictive performance for any new or progressive AKI (area under the receiver operating characteristics curve, 0.81-0.82) and severe AKI (area under the receiver operating characteristics curve, 0.87-0.88) across validation cohorts. Inverse probability weighting provided a reliable and consistent method for handling missing outcome data, ensuring accurate risk estimates for both hospitalized and discharged patients. Models performed similarly across diverse subgroups and ED sites.

**Conclusion:**

Machine learning models trained on routinely collected ED data can provide reliable early predictions of AKI progression, supporting actionable clinical decision making for a broad spectrum of patients. This study advances the real-world usability of such models by expanding their applicability to discharged patients and by enabling estimation of ongoing kidney risk, irrespective of AKI status on arrival.

**The Bottom Line**

Acute kidney injury is common after emergency department (ED) care and is associated with excess morbidity and mortality, yet short-term kidney risk is difficult to assess during the emergency visit. In more than 1.1 million ED encounters, we developed and validated machine learning models to predict new or worsening kidney injury within 72 hours, with good discrimination (area under the curve 0.81 for any kidney injury and 0.87 for severe injury). By enabling prediction-driven, kidney-focused clinical decision support during emergency care, these models have the potential to inform earlier interventions and improve patient outcomes.

## 1 Introduction

### 1.1 Background

Acute kidney injury (AKI) is a prevalent clinical syndrome directly linked to increased morbidity and mortality. Patients with AKI face heightened risks of prolonged and costly hospital stays, progression to chronic kidney disease, dialysis, major adverse cardiovascular events, and death.^1^, ^2^, ^3^, ^4^ Outcomes can be improved through early recognition and optimization of care.^5^, ^6^, ^7^, ^8^ Currently, diagnosis of AKI depends on detecting elevated serum creatinine (sCr) concentration or reduced urine output^9^; both markers are indirect indicators of kidney function and can lag days behind the onset of kidney injury, contributing to underrecognition and delayed diagnosis, especially in emergency department (ED) settings. As a result, emergency clinicians frequently make high-impact decisions regarding medications, diagnostic testing, fluid management, and disposition without reliable insight into near-term kidney risk. Prior ED-based studies have demonstrated frequent exposure to nephrotoxic medications, delayed recognition of AKI, and substantial variability in adherence to kidney-protective practices, underscoring a critical opportunity for early, decision-support–driven intervention during emergency care.^10^^,^^11^

### 1.2 Importance

Application of artificial intelligence (AI) to routinely collected clinical data holds promise for AKI prediction and prevention.^10^ Several groups, including our own, have reported on the development of machine learning (ML) models that uncover subtle patterns in electronic health record (EHR) data to generate reliable AKI risk estimates in hospitalized patients.^11^, ^12^, ^13^, ^14^ These models are well suited to supporting individualized clinical decision support (CDS) by identifying patients who may benefit from kidney-protective interventions before overt injury occurs. Because care trajectories for >140 million patients are set in EDs across the country each year, the potential impact of prediction-driven CDS in emergency care is substantial.^15^^,^^16^

To be used in the ED, AI-informed CDS must be broadly applicable with high reliability across a wide range of clinical presentations. Unfortunately, in the episodic care environment of the ED, the development of ML models to drive such decision support is challenged by missing outcome data. Although the EHR facilitates automated capture of predictor variables and clinical outcomes in ED patients admitted to the hospital, most ED patients are discharged to the community, where outcomes go unmeasured.^15^ Common approaches to address this challenge are to exclude patients with missing outcomes data from model development cohorts (complete case analyses) or to assume negative outcomes in those for whom follow-up data are unavailable.^12^^,^^17^, ^18^, ^19^, ^20^ The first approach results in models that are trained to make reliable predictions in ED patients who are ultimately hospitalized but are completely naive to the larger proportion of patients who are discharged, seen in the ED. The second approach may result in overestimation of model performance during development and underestimation of risk across the population when deployed in real time, limiting their ability to safely inform individualized CDS across the full ED population.

### 1.3 Goals of This Investigation

In this study, we sought to develop a series of AKI prediction models that could integrate seamlessly into ED clinical workflows, providing early, actionable risk estimates that support both disposition decisions and timely AKI mitigation interventions. Specifically, we intended to enable prediction outputs that can be linked to patient-specific clinical actions, such as medication review, nephrotoxin avoidance, diagnostic planning, and decisions regarding monitoring or hospitalization. Unlike previous efforts, which have primarily focused on hospitalized patients, we explicitly account for the challenges of missing outcomes data to expand applicability to discharged patients—an attribute required for utility in the ED, where decision support is needed long before disposition has been determined. Here, we describe the derivation and validation of ML models that estimate risk for new or progressive AKI within 72 hours of ED departure, underscoring their potential to improve patient outcomes and advance real-time CDS in emergency care.

## 2 Methods

### 2.1 Setting and Selection of Participants

This retrospective study was performed using a cohort of patient visits from 5 EDs within the Johns Hopkins Health System. This included 2 urban academic EDs (Johns Hopkins Hospital [JHH] and Johns Hopkins Bayview Medical Center [BMC]), an urban community ED (Sibley Memorial Hospital [SMH]), and 2 suburban community EDs (Howard County Medical Center [HCMC] and Suburban Hospital [SH]). Adult patients (aged ≥18 years) who visited a study site ED between January 1, 2017, and May 31, 2024, and had a metabolic panel that included sCr performed during their ED encounter were included. Patients with end-stage kidney disease, kidney replacement therapy dependence, or a baseline sCr concentration ≥4.0 mg/dL and those who already met criteria for severe AKI (stage 3)^9^ at ED presentation were excluded. Each ED encounter was treated as a distinct index visit, including repeat visits by the same patient, to reflect the clinical reality that risk assessment and decision making occur independently at each presentation.

### 2.2 Data Use and Ethics Oversight

Clinical data used in this study contain protected health information, and their use is governed by the Johns Hopkins Medicine Data Trust and Institutional Review Board (IRB). All analyses described in this study were performed within a HIPAA-compliant computing environment. The study was approved by the Johns Hopkins Medicine IRB under a waiver of informed consent based on a minimal risk determination (IRB Number: IRB00125114).

### 2.3 Methods of Measurement

Outcome and predictor data were extracted from a relational database that underpins the EHR (Epic, Verona, WI) used at all 5 study sites. The prediction timepoint for each index ED visit was defined as the time at which the first serum metabolic panel resulted during that visit, consistent with when early management and disposition decisions are typically made in the ED. To be included, predictor data had to be recorded and available in the EHR prior to this time point.

### 2.4 Outcome and Predictor Variables

#### 2.4.1 Outcomes

The primary predicted outcome in this study was any new or progressive AKI within 72 hours of ED departure. Acute kidney injury was defined and staged according to Kidney Disease Improving Global Outcomes (KDIGO) sCr-based criteria: stage 1 AKI—absolute increase of ≥0.3 mg/dL or ≥1.5 times baseline; stage 2 AKI—increase of 2.0-2.9 times baseline; stage 3 AKI—increase to ≥4.0 mg/dL or ≥3.0 times baseline or initiation of RRT.^9^ Urine output-based criteria were not included in our definition of AKI because this variable was not reliably recorded in the EHR. Baseline sCr was defined as the median of all sCr measurements for the patient in the 180 days prior to the ED encounter or as the sole sCr measurement in the 180 days prior to the ED encounter if only 1 measurement was recorded.^21^ If baseline sCr was unavailable, the expected baseline was estimated using the chronic kidney disease-EPI formula.^22^ For each patient, the initial AKI stage (0 [no AKI], 1, 2, or 3) was determined by comparing the first sCr measured during the index ED encounter against baseline sCr. Peak sCr within 72 hours of ED departure was compared with baseline to determine the highest AKI stage met during the outcome window. Outcomes were flagged as positive if the peak AKI stage exceeded the initial stage, indicating AKI progression or the finding of any stage of new AKI. Separate models were also developed to predict new or progressive AKI that met KDIGO criteria for moderate (stage 2) or severe (stage 3) AKI. Follow-up sCr measurements used to ascertain outcomes were captured from any clinical encounter within the Johns Hopkins Health System, including ED revisits, inpatient admissions, or outpatient laboratory testing occurring within 72 hours of ED departure. If no follow-up sCr was obtained within 72 hours of the patient’s ED departure, the outcome was flagged as missing, and cases were treated as described below.

#### 2.4.2 Predictors

Data for prediction were confined to those routinely collected and stored in the EHR during ED care delivery. Predictor variables were selected based on clinical relevance and availability at the time of ED decision making, and models were trained to optimize predictive performance rather than to estimate causal effects of individual variables. Predictors included patient demographics, comorbidities, chief complaints, initial triage vital signs, routine laboratory results, historical or imputed baseline sCr, and initial AKI stage. Demographic data were restricted to age in years and sex. Comorbidities were identified using ICD-10 codes. Chief complaints were grouped into clinically meaningful categories using a previously defined schema.^23^ Triage vitals included systolic and diastolic blood pressure, temperature, heart rate, respiratory rate, and oxygen saturation. Included laboratory results were serum or plasma albumin, anion gap, blood urea nitrogen, sCr, glucose, potassium, sodium, lactate, troponin, white blood cell count, hemoglobin, platelets, and calculated anion gap and urine-specific gravity. Vitals and laboratory results were further processed to the most recent, minimum, and maximum values obtained before, or concurrently with, the first metabolic panel.

### 2.5 Analytical Methods

#### 2.5.1 Predictive model development

Predictive models were derived and evaluated out-of-sample within the subcohort of ED encounters that occurred between 2017 and 2022. Separate extreme gradient boosting [XGBoost]) algorithms were trained to predict each outcome (ie, any new or progressive AKI, and moderate or severe AKI).^24^ XGBoost is an ensemble decision tree learning model that leverages sequential gradient descent optimization across decision tree iterations to minimize the loss function and improve prediction accuracy while also using regularization techniques to avoid overfitting. XGBoost handles missing predictor data natively using a sparsity-aware split finding algorithm, making it particularly suitable for prediction in contexts where variability exists in the collection of predictor data.^24^ XGBoost hyperparameter optimization was conducted using a grid search to determine the optimal settings, including the subsample ratio of training instances, regularization terms, number of estimators, maximum depth, and learning rate parameters for column subsampling.

#### 2.5.2 Missing predictor data

Missing categoric data (ED arrival mode, sex, chief complaint, and comorbidities) were assigned to a “null” category. Missing continuous values (eg, age, laboratory values, vital signs) were retained as null and handled natively by XGBoost.^24^ If baseline sCr was missing, the estimated baseline was calculated as described above in the Outcomes section and this imputed value was used as baseline.

#### 2.5.3 Missing outcome data

We implemented and compared 4 methods for handling missing outcome data.

1. Incomplete case exclusion: Only complete cases (those where repeat sCr was measured within 72 hours of ED departure) were included in the model training cohort.
2. Negative outcome assumption: All cases were included in training, and cases where sCr was not measured within 72 hours of ED departure were assumed to be outcome negative (no new or progressive AKI).
3. Multiple imputation: All cases were included in training, but observations from complete cases were used to impute outcomes for incomplete cases. Under this approach, a preliminary XGBoost model was first trained to estimate AKI outcome probability using data from complete cases, then employed to generate an outcome probability for each incomplete case. Probabilities were used to impute 20 outcomes per incomplete case, and these outcomes were randomly sampled to generate a fully complete case training cohort with equal representation for all encounters.
4. Inverse probability weighting: Only complete cases were included in training, but each complete case was weighted based on the likelihood of outcome missingness. This approach enabled pseudorepresentation of incomplete cases; complete cases in which the missingness of outcome data was expected (but not observed) were given more weight than cases in which the presence of outcome data was expected. Using the entire data set (complete and incomplete cases), a separate XGBoost model was developed to estimate the likelihood of outcome missingness for each case. This model was used to assign propensity scores to all complete cases, and these propensity scores were then incorporated into our AKI prediction models.

Multiple imputation and inverse probability weighting were employed under the assumption that outcomes are missing at random.^25^^,^^26^ Throughout, “missing at random” is used in its formal causal inference sense—that outcome availability may depend on observed clinical variables but not on unobserved outcomes conditional on those variables—rather than implying random or unpredictable follow-up. The decision to perform repeat sCr measurement within 72 h of ED departure, whether in the inpatient or outpatient setting, is based on individual patients’ medical history, baseline and current kidney function, and general markers of illness severity (eg, vital signs, laboratory values). Each of these factors is included in our prediction model training process.

#### 2.5.4 Performance assessments

The performance of the 4 models developed using the methods described above was evaluated using 2 separate approaches.

First, a standard out-of-sample prediction performance evaluation was performed under a 10-fold cross-validation framework, wherein repeated rounds of training and testing were employed to ensure that all eligible encounter data were used for model training and internal validation (out-of-sample testing) and to limit overfitting.^27^ Models were then externally validated in a second set of ED encounter data, separated in time from those used for development. Regardless of the method used for model training, only ED visits with observed outcomes were included in test set evaluations. Receiver operating characteristic curve analysis (with CIs and performance differences determined by DeLong’s method), precision recall curve analysis to assess diagnostic performance across a range of thresholds, calibration curves to plot observed versus predicted risk, and the Brier score for overall goodness of fit were all performed.

Second, the relative performance of models developed under each method in predicting AKI was assessed by comparing the mean predicted probabilities of AKI with the *observed* outcome rate for complete cases and the *expected* outcome rate for incomplete cases. To derive an expected AKI outcome rate for incomplete cases, we relied on previously published relative frequencies of new or progressive chronic kidney disease within 3 months of ED visits that resulted in discharge or hospitalization at our study sites (1.8% and 5.0%, respectively; ratio 0.36).^4^ Chronic kidney disease is known to be highly correlated with AKI,^28^ and most patients in our cohort with missing AKI outcomes were discharged from the ED. Thus, we estimated that the rate of AKI in cases where repeat sCr concentration was not measured within 72 hours of ED departure (outcome missing) would be 36% of that observed in cases where repeat sCr concentration was measured during the outcome window.

Model interpretation was facilitated through feature importance measures, including SHapley Additive exPlanations (SHAP) values, to assess the impact and directionality of predictors.

#### 2.5.5 Sensitivity analyses

To assess the possible influence of missing outcomes (incomplete cases) on area under the curve (AUC) measures, we also performed sensitivity analyses using the approach of Rotnitzky, Faraggi, and Schisterman.^29^ This approach assumes a specific (unverifiable) form of outcome missingness, namely, the log likelihood of having the outcome observed is a function of underlying covariates plus a constant offset (*μ*, if the patient has AKI regardless of whether it is observed; zero, otherwise). We use the same propensity model functional form and evaluate *μ* over a range of values (−1 to 3) to capture different missingness scenarios (with *μ*>0 corresponding to increased odds of the outcome being observed for those with AKI compared with those without AKI, conditional on other features). In addition to individual AUC estimates (with bootstrapped CIs) for each of the 4 models, we also do pairwise comparisons (for a given value of *μ*) relative to the complete case model. All results are included in the supplemental material.

#### 2.5.6 Software

All model building and statistical analyses were conducted using Python 3.10.11, with essential packages from scikit-learn version 1.4.0, XGBoost version 2.0.3, and SHAP version 0.45.1. Missingness sensitivity analyses were conducted in R version 4.4.1. ChatGPT (OpenAI) was used to assist with framing and refining the manuscript. The tool did not contribute to study design, data analysis, or interpretation, and all content was reviewed, edited, and finalized by the authors, who take full responsibility for the content.

## 3 Results

**Figure 1:**
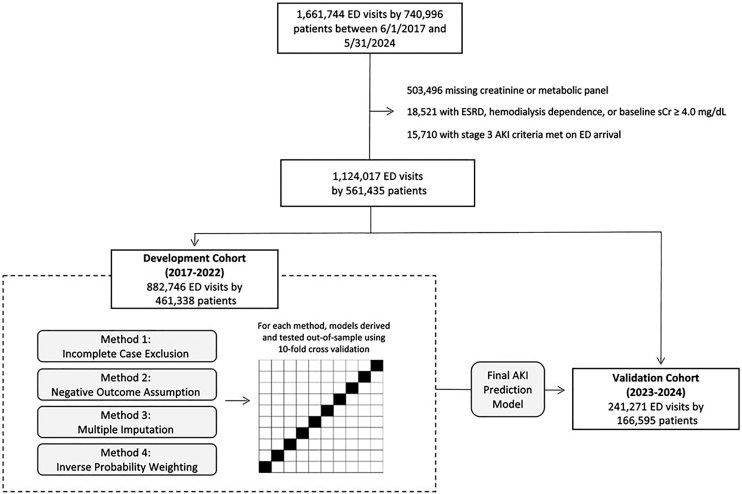
Study flowchart. Four sets of prediction models were developed using varied methods to account for missing outcomes data. Under a 10-fold cross-validation approach, all data in the development cohort, comprised emergency department (ED) encounters at all 5 study sites between June 1, 2017, and December 31, 2022, were used for both derivation and out-of-sample testing of each model. Models were then externally validated using data from a future time period (January 1, 2023-May 31, 2024). AKI, acute kidney injury; ED, emergency department.

There were 1,661,744 ED visits by 740,996 patients during our study period. After applying exclusion criteria, our final study cohort included 1,124,017 ED visits by 561,435 patients (Fig 1). Visits that occurred between 2017 and 2022 (*n* = 882,746; 78.5%) were allocated to our model development cohort; those that occurred in 2023 and 2024 (*n* = 241,271; 21.5%) were held aside for external validation of our final prediction models.

### 3.1 Cohort Characteristics

Development and validation cohorts included representation across age groups, sex, race, and ethnicity (Table 1).^21^^,^^22^ Median baseline sCr concentration (1.1 mg/dL) and overall prevalence and severity of AKI (7.1% with stage 1 AKI and 1.4% with stage 2 AKI) at presentation were consistent across the entire study population. Approximately 40% of patients were hospitalized after their ED encounter, whereas the remainder were discharged into the community. Repeat sCr concentration was measured within 72 hours of ED departure in 34.5% of visits, with AKI observed during the outcome window for 5.7% of these visits (4.1%, stage 1; 1.0%, stage 2; 0.6%, stage 3). Rates of hospitalization, repeat sCr measurement, AKI outcome incidence, and AKI outcome severity were similar in development and validation cohorts (Table 1). AKI trajectory across the entire study cohort is further depicted graphically in Figure S1. Rates of AKI, both AKI present on arrival and progression of AKI during the outcome window, were slightly higher at our 2 academic ED study sites; other patient characteristics, including demographics and hospitalization rates, were similar across sites (Table S1).

**Table 1:** Study cohort characteristics.

Patient characteristic	Total	Development	Validation
Total visits	1,124,017	882,746	241,271
Age subgroup, *N* (%)			
18-44 y	424,105 (37.7)	333,223 (37.7)	90,882 (37.7)
45-64 y	340,808 (30.3)	267,453 (30.3)	73,355 (30.4)
65-74 y	153,201 (13.6)	120,392 (13.6)	32,809 (13.6)
>74 y	205,903 (18.3)	161,678 (18.3)	44,225 (18.3)
Sex at birth, *N* (%)			
Female	618,866 (55.1)	485,969 (55.1)	132,897 (55.1)
Race/ethnicity, *N* (%)			
Black non-Latino	397,630 (35.4)	312,878 (35.4)	84,752 (35.1)
White non-Latino	531,852 (47.3)	417,267 (47.3)	114,585 (47.5)
Latino	89,339 (7.9)	69,966 (7.9)	19,373 (8.0)
Other	105,196 (9.4)	82,635 (9.4)	22,561 (9.4)
Baseline serum creatinine, mg/dL (95% CI)	1.1 (0.6-1.7)	1.1 (0.6-1.7)	1.1 (0.6-1.7)
AKI stage on arrival, *N* (%)			
None	1,028,681 (91.5)	807,874 (91.5)	220,807 (91.5)
Stage 1	79,867 (7.1)	62,763 (7.1)	17,104 (7.1)
Stage 2	15,469 (1.4)	12,109 (1.4)	3360 (1.4)
ED disposition, *N* (%)			
Discharged	677,105 (60.2)	527,366 (59.7)	149,739 (60.1)
Hospitalized	446,912 (39.8)	355,380 (40.3)	91,532 (37.9)
New or progressive AKI within 72 h, *N* (%)			
Repeat creatinine not measured within outcome window	736,518 (65.5)	576,478 (65.3)	160,040 (66.3)
Repeat creatinine measured within outcome window	387,499 (34.5)	306,268 (34.7)	81,231 (33.7)
No progression, *N* (%)	365,406 (94.3)	288,585 (94.2)	76,821 (94.6)
Progression, *N* (%)	22,093 (5.7)	17,683 (5.8)	4410 (5.4)
Peak stage 1, *N* (%)	15,825 (4.1)	12,706 (4.1)	3119 (3.8)
Peak stage 2, *N* (%)	3954 (1.0)	3140 (1.0)	814 (1.0)
Peak stage 3, *N* (%)	2314 (0.6)	1837 (0.6)	477 (0.6)

AKI, acute kidney injury; ED, emergency department.

Baseline serum creatinine was defined as the median of all measurements for the patient in the 180 days prior to the visit or as the sole serum creatinine measurement if only 1 was recorded.^21^ If baseline creatinine was unavailable, the expected baseline was estimated using the CKD-EPI formula.^22^ AKI was staged using Kidney Disease Improving Global Outcomes serum creatinine-based criteria, and comparison of the current creatinine to the preencounter baseline.

### 3.2 Model Specification and Predictive Performance

Final models contained 131 distinct prediction variables, comprising demographics (excluding race and ethnicity), baseline kidney function testing results, ED chief complaints, comorbidities, ED vital signs, and ED laboratory results. Predictor values were similar in the development and validation cohorts (Table S2). Overall prediction performance, as measured using area under the receiver operating characteristic analysis, was similar in the subset of patients with observed outcomes regardless of the method employed to account for outcome missingness during training (Table 2). Under all training scenarios, model accuracy was higher for the prediction of progression to severe AKI (AUC, 0.87-0.88) than for any-severity AKI (AUC, 0.81-0.82). For both outcomes, out-of-sample prediction performance during external validation was comparable to that achieved out-of-sample during development (Table 2). Although specific AUC values were sensitive to missingness assumptions (per sensitivity analyses, see Tables S3 and S4), the relative performance of models was comparable. Predictive performance was also stable when stratified by individual ED study site, sex, reported race, and ethnicity (Table S5).

**Table 2:** Prediction performance of models trained using varied methods to account for missing outcomes.

Predicted outcome	Model training method	Development cohort	Validation cohort
Any new or progressive acute kidney injury	Complete case training	0.82 (0.82-0.82)	0.82 (0.81-0.82)
	missing outcomes assumed negative	0.80 (0.80-0.81)	0.80 (0.80-0.81)
	Multiple imputation	0.82 (0.81-0.82)	0.82 (0.81-0.82)
	inverse probability weighting	0.81 (0.81-0.82)	0.81 (0.81-0.82)
Progression to severe acute kidney injury (stage 2 or 3)	Complete case training	0.88 (0.87-0.88)	0.87 (0.86-0.88)
	missing outcomes assumed negative	0.88 (0.87-0.88)	0.87 (0.86-0.88)
	Multiple imputation	0.88 (0.86-0.88)	0.87 (0.86-0.89)
	inverse probability weighting	0.88 (0.87-0.88)	0.87 (0.86-0.88)

Differences in model performance did emerge when comparing mean predicted probabilities to observed outcome rates for complete cases and estimated outcome rates for cases where repeat creatinine measurement was not performed within the outcome window (incomplete cases). Within complete cases, the observed rates of AKI and severe AKI were 5.70% and 1.62%, respectively; using the approach described in the Methods section, we derived estimated outcome rates of 2.05% and 0.58% for incomplete cases (Table 3). Worst predictive performance was observed when missing outcomes were assumed negative during training; under this approach, predicted probabilities were lower than expected for both complete and incomplete cases. In contrast, models trained using any of the other 3 approaches (complete case training, multiple imputation, or inverse probability weighting) generated mean predicted probabilities that were similar to both observed and estimated outcome rates (Table 3).

**Table 3:** Observed outcome rates versus predicted probabilities for various modeling techniques.

Predicted outcome	Method	Complete cases (*N* = 81,231)	Incomplete cases (*N* = 160,040)
Any new or progressive acute kidney injury	Outcome rate	5.70% (observed)	2.05% (expected)
	Mean predicted probabilities		
	Complete case training	5.24 (5.19-5.29)	2.42 (2.4-2.44)
	Missing outcomes assumed negative	3.36 (3.32-3.4)	0.88 (0.87-0.89)
	Multiple imputationa	5.2 (5.15-5.25)	2.4 (2.38-2.42)
	Inverse probability weighting	5.04 (4.99-5.09)	2.13 (2.11-2.15)
Progression to severe acute kidney injury (stage 2 or 3)	Outcome rate	1.62% (observed)	0.58% (expected)
	Mean predicted probabilities		
	Complete case training	1.44 (1.41-1.47)	0.4 (0.39-0.4)
	Missing outcomes assumed negative	1.05 (1.02-1.07)	0.18 (0.17-0.18)
	Multiple imputation	1.43 (1.4-1.45)	0.39 (0.38-0.4)
	Inverse probability weighting	1.39 (1.37-1.42)	0.35 (0.34-0.35)

Mean predicted probabilities are shown for each model and outcome with 95% CIs in parentheses. Expected outcome rates were estimated in incomplete cases based on the previously reported relative outcome rate for chronic kidney disease at 3 months in the same emergency department population (0.36).^21^

**Figure 2:**
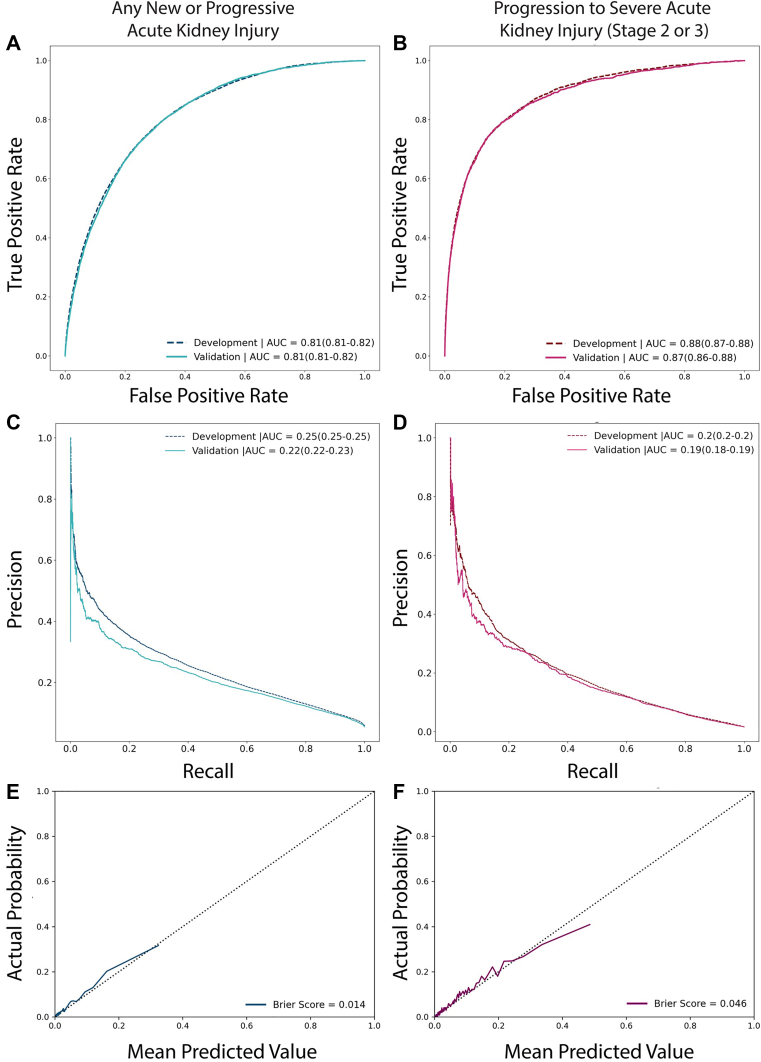
Model performance assessment. Receiver operating characteristic curves (A, B), precision recall curves (C, D), and calibration curves (E, F) are shown for models trained to predict any new or progressive acute kidney injury (left side panels, blue lines) and progression to severe (stage ≥2) acute kidney injury. Receiver operating characteristic and precision recall curves are shown for both development and validation cohorts, whereas actual versus mean predicted probabilities with Brier scores are shown for area under the curve (AUC).

In the absence of large performance differences between these approaches to training, models developed using the simplest method to explicitly account for missing outcomes (inverse probability weighting) were selected for further performance assessment and characterization in the external validation cohort. Receiver operating characteristic and precision recall curves with operating points overlaid across a range of prediction thresholds for both AKI and severe AKI are shown in Figure 2. Binary performance measures, which provide an assessment of end-user–perceived diagnostic performance and vary depending on the selected threshold, are also shown (including sensitivity, specificity, positive and negative predictive values, and *F*1 scores) in Figure S3. For example, if a risk threshold of 10% were chosen for labeling patients as high risk for progressive AKI within 72 hours, approximately 14% of patients would be labeled as high risk, sensitivity would be 50.82%, specificity would be 88.03%, and labels would have positive and negative predictive values of 19.59% and 96.89%, respectively. Increasing the risk threshold to 40% would decrease the frequency of labels to less than 1% and increase the positive predictive value to 41.8% but lower the sensitivity to 5.15%. Similar scenarios are shown for labeling as high risk for severe AKI.

As shown in Figure S2, markers of baseline and current kidney function (creatinine concentration and stage of AKI at ED arrival) were among the most influential factors in both prediction models. Interestingly, a history of cerebrovascular disease was also an important predictor and was the strongest predictor of our primary outcome. Age, ED vital signs, and several laboratory parameters, including serum glucose, albumin, white blood cell count, and urine-specific gravity, were also important predictors.

## 4 Limitations

Despite its strengths, this study has several limitations. First, although we employed multiple methods to address missing outcome data, the accuracy of these approaches relies on assumptions that may not fully capture the true nature of outcome missingness. Second, our reliance on serum creatinine-based KDIGO criteria excludes urine output as an outcome measure, potentially underestimating AKI incidence.^9^^,^^30^ This limitation is difficult to avoid, as urine output is not routinely measured in ED settings. This reliance also led to exclusion of encounters where creatinine was not ordered in the ED; although this may limit generalizability, patients would not be candidates for AKI-focused decision support without an initial assessment of kidney function (eg, creatinine measurement). Third, the study was conducted within a single health system, which may limit generalizability to other settings with different patient populations or clinical practices.^13^ Although this limitation was mitigated through the inclusion of a diverse set of EDs within this system (and preserved prediction performance across sites), it remains possible that system-specific factors could have affected performance. Finally, as an observational prediction study, this work does not assess whether use of model-informed CDS improves clinical outcomes; a prospective evaluation will be required to determine how best to integrate predictions into ED workflows and measure downstream impact. To date, personalized strategies for management of early AKI have had mixed success; it will be important to understand whether such interventions can be more effective when paired with predictive technology deployed very early in the hospital encounter.^5^, ^6^, ^7^, ^8^^,^^31^, ^32^, ^33^

## 5 Discussion

In this study, we developed and validated a series of ML models to predict new or progressive AKI using clinical data collected from diverse EDs within a large health system. By design, these models are intended to support individualized, kidney-focused CDS at the point in emergency care when key management decisions are made. Early risk estimates generated during the ED visit can inform medication selection, nephrotoxin avoidance, diagnostic planning, monitoring intensity, and disposition—all decisions that are made before inpatient teams are involved, often using incomplete information and under significant time pressure.^34^, ^35^, ^36^ Prior studies have demonstrated that AKI and adverse downstream events occur commonly in ED populations, that these outcomes vary with ED-based decision making, and that opportunities exist to optimize kidney care in the ED setting.^4^^,^^37^, ^38^, ^39^, ^40^, ^41^

In contrast to models that operate across an entire hospitalization,^13^^,^^14^^,^^42^ we intentionally limited ours to predictor data accumulated within hours of presentation to the health-care system, and all predictions were made at the time of initial kidney function assessment. This is the timepoint most pertinent for emergency clinicians’ decisions—and most important for patients—as these early decisions set care trajectories. Despite reliance on a more restricted set of predictors, the accuracy of our models meets or exceeds that reported for many others, including those that are commercially available.^14^^,^^42^, ^43^, ^44^ The utility of our prediction models may be best appreciated by examining the performance curves shown in Figure 2 and binary classification measures shown in Figure S3, whereas considering how these models could be used to inform real-time patient-centered CDS. For example, the observed prevalence of severe AKI at 72 hours was 1.4% in our study cohort, yet the area under the precision recall curve of our severe AKI prediction model was 19%, indicating a 13.5-fold improvement over chance alone. By way of comparison, the improvement over chance reported for many other prediction models is more modest, in the 3- to 5-fold range.^14^^,^^42^^,^^44^ If incorporated into a CDS system where initial kidney function labs were accompanied by gradations of near-term AKI risk, our model could inform a highly selective high-risk label (applicable to 3 in 100 patients) that would indicate a 1 in 5 chance of developing severe AKI within 72 hours.

Another distinguishing feature of our approach is the integration of surveillance and prediction. Prior to generating a prediction, our algorithms surveil the EHR to calculate each patient’s baseline kidney function and to determine whether they currently meet consensus criteria for AKI.^9^^,^^21^ In cases where historical data are not available, we are able to determine whether current kidney function is below that expected based on age and sex.^22^ By employing a composite outcome for prediction (new or progressive AKI), we are able to estimate ongoing risk for all patients, irrespective of AKI status at arrival. This combined approach enables emergency clinicians to act promptly on existing AKI that may otherwise go unnoticed while also anticipating potential deterioration of kidney function. This dual functionality is important, as prior research has shown that easily diagnosable AKI is very often overlooked and undertreated in both inpatient and emergency care settings.^38^^,^^45^^,^^46^

A third distinguishing feature of our approach is explicit consideration and inclusion of discharged patients. Nationally, up to 85% of all ED encounters result in discharge.^15^ Previously published models, including our own, were trained to make reliable predictions in patients who are hospitalized after their ED encounter, but remained naive to the much larger group discharged into the community.^12^^,^^43^ From a clinical perspective, models that fail to account for discharged patients cannot safely support decision making in the ED, where disposition is often unknown at the time of prediction and kidney-relevant decision making (eg, diagnostic evaluation and medication prescribing). Our modeling approach enables reliable predictions across the full spectrum of ED encounters and expands applicability to all of the approximately 140 million ED encounters that occur in the United States annually.^15^

Finally, a related contribution of this work is the systematic evaluation of methods to address missing outcome data,^20^ a pervasive challenge in ED-based predictive modeling. Our comparison of 4 approaches—incomplete case exclusion, negative outcome assumption, multiple imputation, and inverse probability weighting revealed that methods explicitly accounting for missingness yielded predicted probabilities more consistent with observed and estimated outcome rates. Notably, inverse probability weighting emerged as a practical and effective solution, enabling robust model training while preserving performance across the development and validation cohorts. This methodological rigor underscores the importance of addressing missing outcomes to enhance the reliability and fairness of ML models in clinical practice.

This study has practical implications for the deployment of prediction-based CDS for AKI in the acute care setting. Relying exclusively on predictors routinely available during ED care—including demographic characteristics, chief complaints, vital signs, and laboratory values—our models were able to generate actionable risk estimates early in the clinical workflow. This capability aligns with the recommendations of several national and international organizations to employ consensus-driven AKI mitigation interventions (eg, optimizing volume status, medication review, nephrotoxin avoidance, glycemic control, and nephrology consultation) in a timely fashion for at-risk patients.^9^^,^^47^^,^^48^ Provision of kidney-focused CDS in the ED is important for both high and low-severity patients; emergency clinicians now establish treatment plans for a majority of hospitalized patients^16^^,^^49^ and provide definitive care to the larger number discharged into the community.^15^ Moreover, our model’s transparency, facilitated through SHAP values, provides clinicians with interpretable insights into the factors driving individual predictions. This is a feature that has been identified as essential for fostering trust and promoting adoption of ML tools in high-stakes clinical environments like the ED.^50^^,^^51^

## 6 Conclusion

This study demonstrates the feasibility and utility of ML for AKI prediction in ED settings, addressing key challenges such as missing outcome data and incorporating a diverse population of clinical encounters resulting in either hospitalization or discharge from the ED. By enabling early, individualized estimation of AKI risk across both hospitalized and discharged patients, this work advances the development of prediction-driven CDS tailored to the realities of emergency care, where timely kidney-focused interventions have the greatest potential to influence patient trajectories.

## Author Contributions

Conceptualization and methodology: JH, EK, SL, IS, MC, ME, SM, CP, AS, OBM; Project administration: JH, EK, SL; Supervision: JH, EK; Data curation, analysis, and visualization: XZ, EK, MC; Writing—original draft: JH, XZ, MC; Writing—review and editing: All authors; Resources: JH, GK, EK; Funding acquisition: JH, EK, SL. EK and XZ had full access to all the data in the study. During the preparation of this work, JH used ChatGPT 4.0 to assist with proofreading and to generate suggested edits to enhance readability and clarity of message; all comments and edits were subsequently reviewed and revised by the authors. EK takes responsibility for the integrity of the data and the accuracy of the data analyses. JH takes responsibility for the content of the manuscript as a whole.

## Funding and Support

Data infrastructure used to facilitate this work was built with the support of grant number R18HS026640 from the 10.13039/100000133Agency for Healthcare Research and Quality (AHRQ), 10.13039/100000016U.S. Department of Health and Human Services (HHS). Predictive model development and performance evaluation were supported by grant number R01HS027793 from AHRQ. The funders had no role in the design and conduct of the study, and the authors are solely responsible for this document’s contents, findings, and conclusions, which do not necessarily represent the views of the AHRQ or HHS; readers should not interpret any statement in this report as an official position of the AHRQ or HHS.

## Conflict of Interest

The authors declare no competing financial or nonfinancial interests.
