# Supplementary material for: Development and Validation of Machine Learning Models to Identify Emergency Department Patients at Increased Risk of New or Progressive Acute Kidney Injury
Source: J Am Coll Emerg Physicians Open. 2026 Apr 18;7(3):100397. doi: 10.1016/j.acepjo.2026.100397 (PMC13094641; doi:10.1016/j.acepjo.2026.100397)
Supplement: Supplementary Material [file mmc1.docx]

**Supplemental Materials for “Development and Validation of Machine Learning Models to Identify Emergency Department Patients at Increased Risk of New or Progressive Acute Kidney Injury”, by Hinson et al.**

## Methods

**Imputation of Missing Baseline Creatinine Concentration**

To impute missing baseline serum creatinine concentration values, we assumed a baseline eGFR of 75 mL/min/1.73m² for individuals without a documented history of serum Creatinine. We then used the 2009 CKD-EPI creatinine equation, excluding the race coefficient. Specifically, we applied the equation parameters for "White or Other" individuals across all patients, using sex-specific constants (κ = 0.9 and α = –0.411 for males; κ = 0.7 and α = –0.329 for females).

For each age bracket, we selected a median age and inverted the CKD-EPI equation to calculate the sCr value that would correspond to an eGFR of 75 mL/min/1.73m².

| sex | kappa | alpha |
| --- | --- | --- |
| male | 0.9 | -0.411 |
| female | 0.7 | -0.329 |

This is the original imputation table used in the code:

| Age Range | Male | Female |
| --- | --- | --- |
| 18–24 | 1.3 | 1 |
| 25–29 | 1.2 | 1 |
| 30–39 | 1.2 | 0.9 |
| 40–54 | 1.1 | 0.9 |
| 55–64 | 1.1 | 0.8 |
| 65+ | 1 | 0.8 |

We then used the 2009 CKD-EPI formula to replicate imputed sCr values, values look similar to the table above.

| Age | Male | Female |
| --- | --- | --- |
| 21 | 1.34 | 1.06 |
| 27 | 1.3 | 1.02 |
| 34 | 1.25 | 0.98 |
| 47 | 1.15 | 0.91 |
| 59 | 1.08 | 0.85 |
| 75 | 0.98 | 0.77 |

Paper reference: <https://pmc.ncbi.nlm.nih.gov/articles/PMC2763564/>

## Results

**Sensitivity Analyses**

The results of the sensitivity analyses for (out-of-sample) validation AUC under a specific missingness model (described in main text *Methods*) are shown in Tables S3 and S4. For both model outcomes (any new or progressive AKI, or progression to severe AKI), the differences in AUC across the methods are generally negligible across a wide range of missingness parameters μ from –1 to 3. The value μ=0 corresponds to the “ignorable verification” scenario (i.e., no residual selection bias in outcome availability for those with AKI, conditional on available covariates); in this scenario, we see that the estimated AUC values on the entire validation cohort (as opposed to the subset with available outcomes, as reflected in main text *Table 2*) are slightly higher for the new-or-progressive AKI outcome (0.83-0.84 for the any-stage AKI outcome, versus 0.80-0.82 in the observed outcome group) and similar for the severe AKI outcome (0.87-0.88 versus 0.87-0.88).

Not surprisingly, we also observe that AUC values are generally increasing with increasing values of μ. For example, μ=2 corresponds to residual selection bias in favor of those with AKI (with odds of having outcome observed exp(2)≈7.4 times higher for a patient with AKI compared to one without AKI, conditioned on all covariates); in this case, the new-or-progressive AKI models have estimated AUC 0.87-0.88 (compared to 0.80-0.82 in the observed outcome group) and the severe AKI models have estimated AUC around 0.92 (compared to 0.87). This reinforces that specific estimated AUC values are sensitive to both the population under consideration (only patients with observed outcomes versus all patients) and the assumed missingness model, even if the relative performance between models is similar.


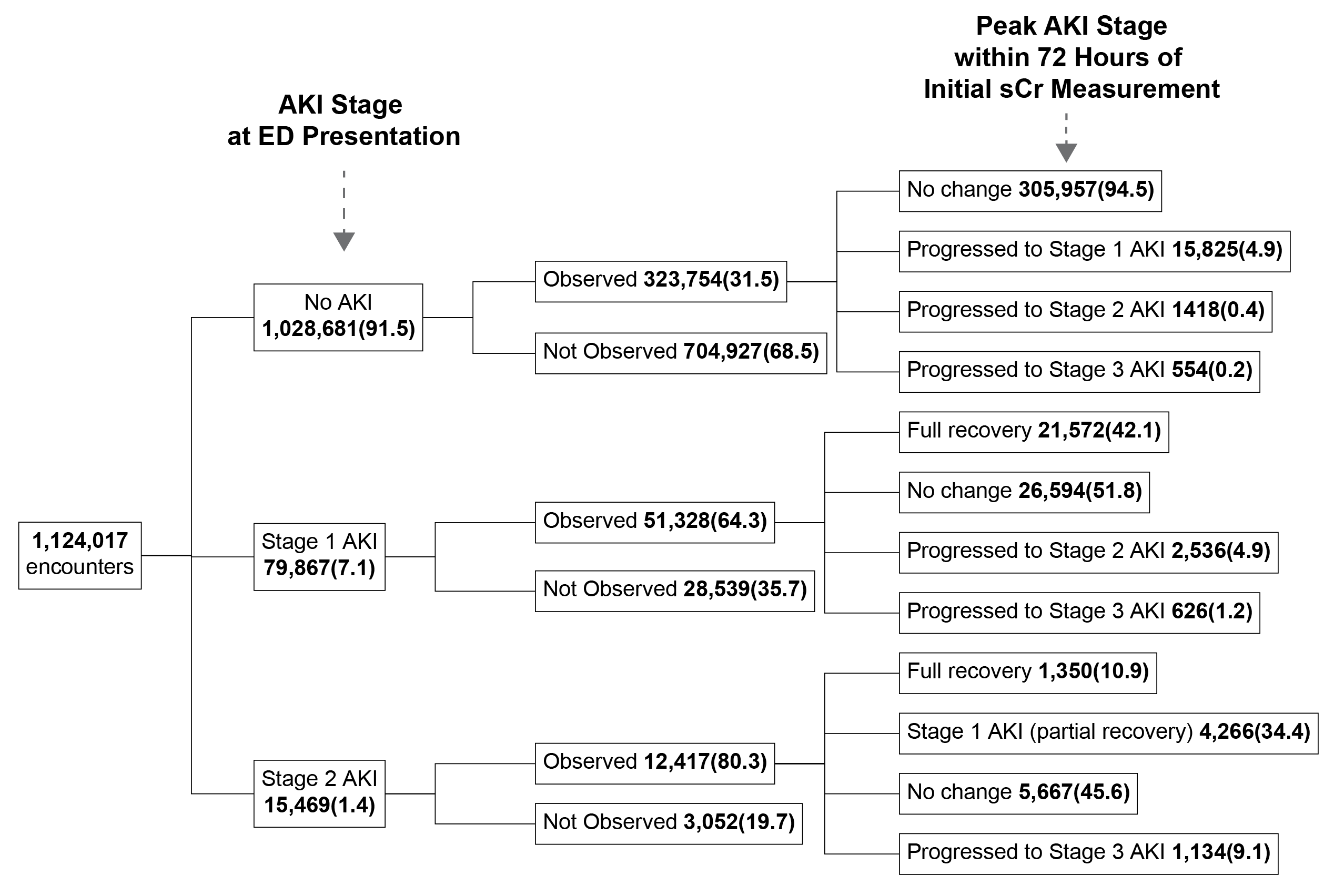


**Figure S1.** Tree diagram visualization of 72-hour kidney function trajectory for all ED encounters. Encounters are shown as frequency counts with percent of immediate parent grouping in parentheses. Encounters where trajectory was observed had at least one repeat creatinine measurement within the outcome window. AKI: acute kidney injury; ED: emergency department; sCr: serum creatinine concentration.

**
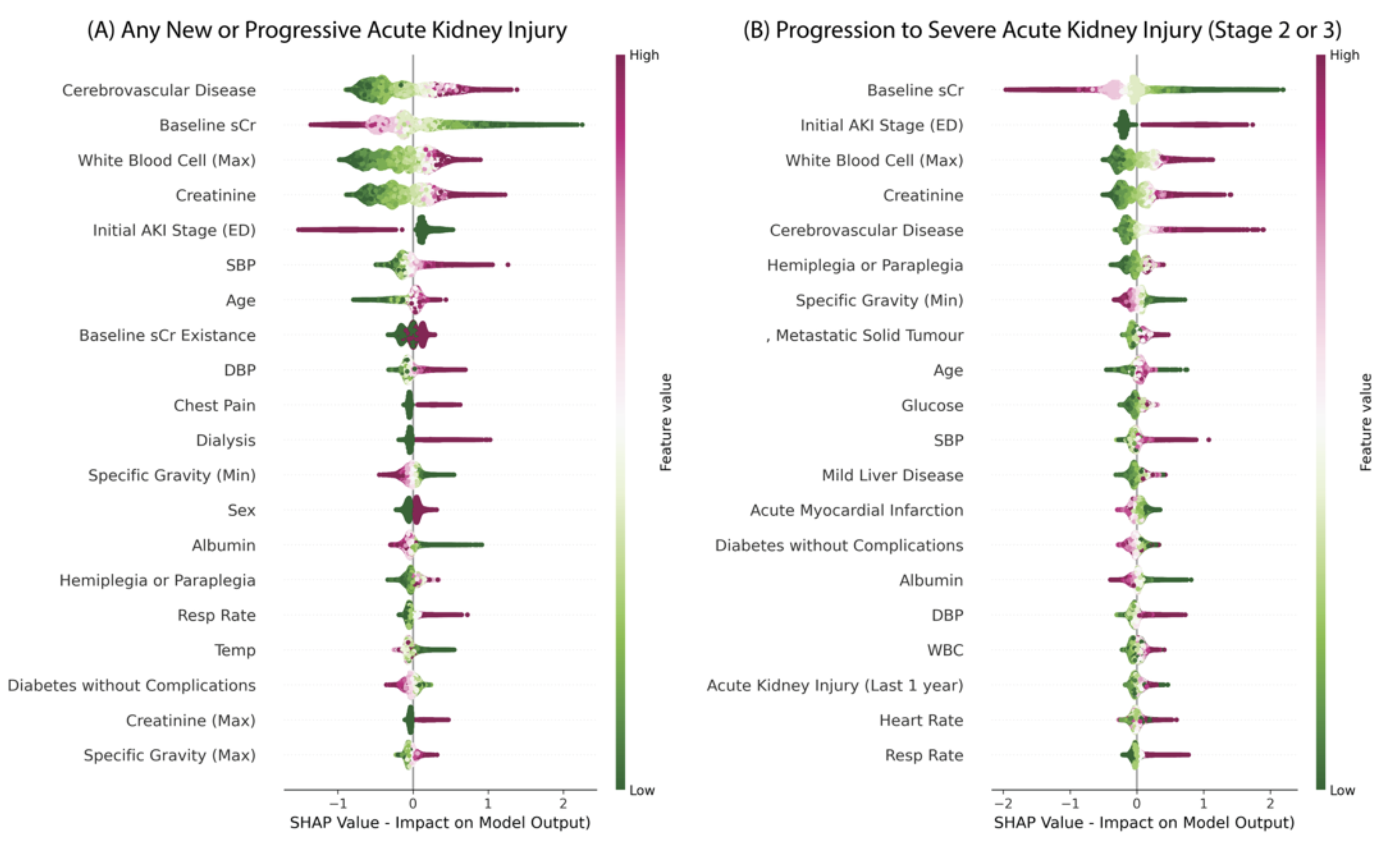
 Figure S2.** Shapley additive explanations (SHAP) values for (A) Any New or Progressive Acute Kidney Injury and (B) Progression to Severe Acute Kidney Injury (Stage 2 or 3) prediction models using Inverse Probability Weighting method


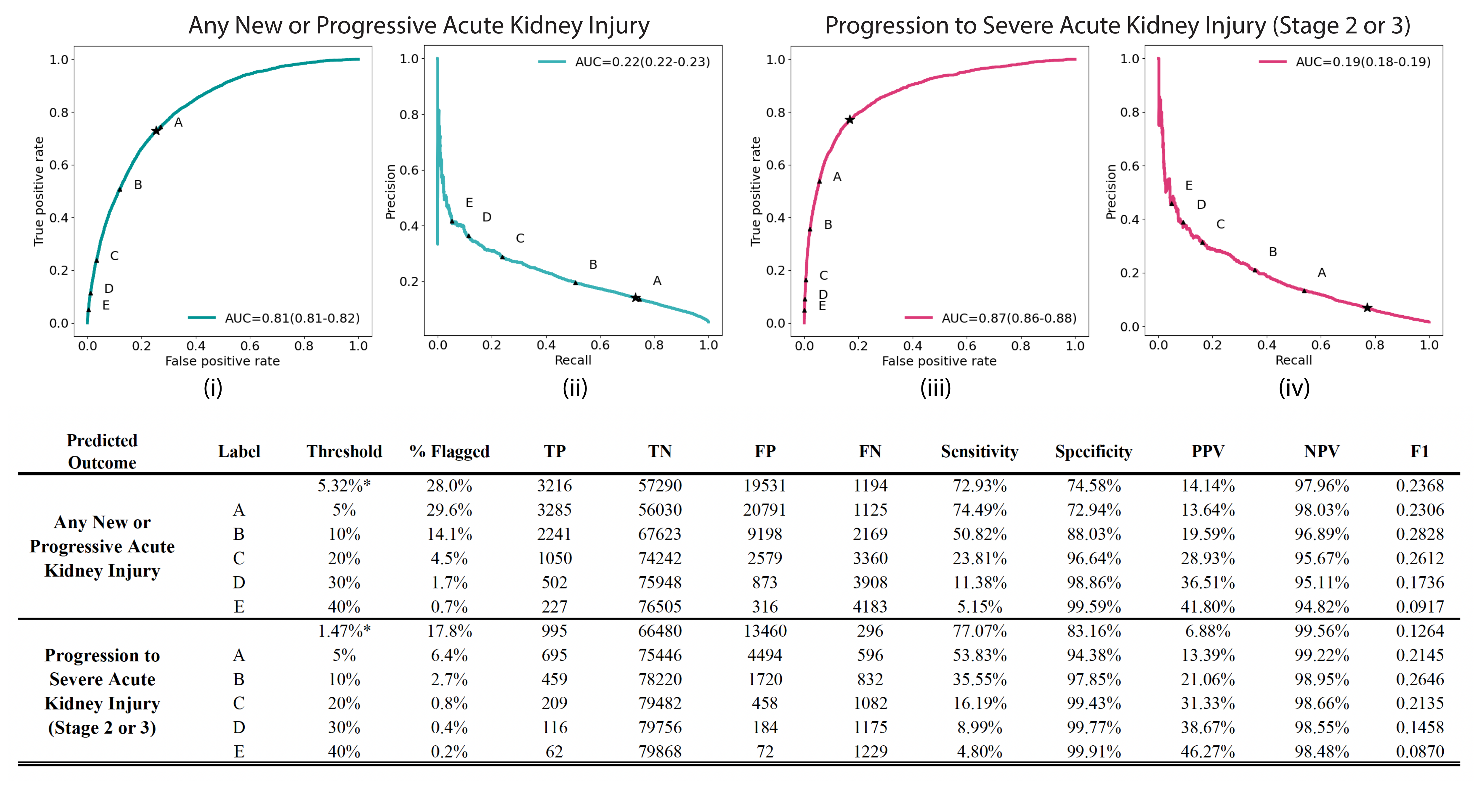
 **Figure S3:** Comprehensive Final Model Performance During Validation. Receiver operating characteristic curves for any new or progressive acute kidney injury (i, ii) and progression to severe acute kidney injury (Stage 2 or 3) (iii, iv) prediction models are reproduced from the main text with operating points across a range of thresholds overlaid. Binary classification measures corresponding to operating points above are shown in the tabular form below. Bold thresholds with corresponding starred operating points represent the point where sensitivity and specificity are simultaneously optimized. FN: false negative; FP: false positive; LR+: positive likelihood ratio; LR-: negative likelihood ratio; NPV: negative predictive value; PPV: positive predictive value; TN: true negative; TP: true positive; * Youden’s Index.

**Table S1. Cohort Characteristics by ED Study Site**

|  | **Total** | **JHH** | **BMC** | **HCGH** | **SH** | **SMH** |
| --- | --- | --- | --- | --- | --- | --- |
| **Total Visits** | 1124017 | 265605 | 210810 | 282320 | 209264 | 156018 |
| **Age Subgroup, N (%)** |  |  |  |  |  |  |
| **18-44 years** | 424105 (37.7) | 121007 (45.6) | 82579 (39.2) | 107424 (38.1) | 58654 (28.0) | 54441 (34.9) |
| **45-64 years** | 340808 (30.3) | 92115 (34.7) | 68817 (32.6) | 82818 (29.3) | 55459 (26.5) | 41599 (26.7) |
| **65-74 years** | 153201 (13.6) | 31254 (11.8) | 28767 (13.6) | 37829 (13.4) | 32577 (15.6) | 22774 (14.6) |
| **>74 years** | 205903 (18.3) | 21229 (8.0) | 30647 (14.5) | 54249 (19.2) | 62574 (29.9) | 37204 (23.8) |
| **Sex at Birth, N (%)** |  |  |  |  |  |  |
| **Female** | 618866 (55.1) | 137564 (51.8) | 109854 (52.1) | 163872 (58.0) | 114115 (54.5) | 93461 (59.9) |
| **Race/Ethnicity, N (%)** |  |  |  |  |  |  |
| **Black non-Latino** | 397630 (35.4) | 154181 (58.0) | 66826 (31.7) | 88216 (31.2) | 36655 (17.5) | 51752 (33.2) |
| **White non-Latino** | 531852 (47.3) | 79565 (30.0) | 114399 (54.3) | 136157 (48.2) | 119338 (57.0) | 82393 (52.8) |
| **Latino** | 89339 (7.9) | 15913 (6.0) | 19360 (9.2) | 21109 (7.5) | 25072 (12.0) | 7885 (5.1) |
| **Other** | 105196 (9.4) | 15946 (6.0) | 10225 (4.9) | 36838 (13.0) | 28199 (13.5) | 13988 (9.0) |
| **Baseline Creatinine Concentration, mg/dL** | 1.1 (0.6-1.7) | 1.1 (0.5-1.9) | 1.1 (0.6-1.8) | 1.1 (0.6-1.6) | 1.1 (0.6-1.6) | 1.1 (0.6-1.5) |
| **Acute Kidney Injury Stage on Arrival, N (%)** |  |  |  |  |  |  |
| **No AKI** | 1028681 (91.5) | 243143 (91.5) | 187858 (89.1) | 259081 (91.8) | 192948 (92.2) | 145651 (93.4) |
| **Stage 1** | 79867 (7.1) | 18929 (7.1) | 19264 (9.1) | 19450 (6.9) | 13607 (6.5) | 8617 (5.5) |
| **Stage 2** | 15469 (1.4) | 3533 (1.3) | 3688 (1.7) | 3789 (1.3) | 2709 (1.3) | 1750 (1.1) |
| **ED Disposition, N (%)** |  |  |  |  |  |  |
| **Discharged** | 677105 (60.2) | 157362 (59.2) | 117928 (55.9) | 170888 (60.5) | 123605 (59.1) | 107322 (68.8) |
| **Hospitalized** | 446912 (39.8) | 108243 (40.8) | 92882 (44.1) | 111432 (39.5) | 85659 (40.9) | 48696 (31.2) |
| **New or Progressive Acute Kidney Injury within 72 hours, N (%)** |  |  |  |  |  |  |
| **Repeat Creatinine Not Measured within Outcome Window** | 736518 (65.5) | 163326 (61.5) | 127558 (60.5) | 195598 (69.3) | 137363 (65.6) | 112673 (72.2) |
| **Repeat Creatinine Measured within Outcome Window** | 387499 (34.5) | 102279 (38.5) | 83252 (39.5) | 86722 (30.7) | 71901 (34.4) | 43345 (27.8) |
| **No Progression, N (%)** | 365406 (94.3) | 96076 (93.9) | 77921 (93.6) | 82094 (94.7) | 67856 (94.4) | 41459 (95.6) |
| **Progression N (%)** | 22093 (5.7) | 6203 (6.1) | 5331 (6.4) | 4628 (5.3) | 4045 (5.6) | 1886 (4.4) |
| **Peak Stage 1, N (%)** | 15825 (4.1) | 4791 (4.7) | 3739 (4.5) | 3185 (3.7) | 2815 (3.9) | 1295 (3.0) |
| **Peak Stage 2, N (%)** | 3954 (1.0) | 914 (0.9) | 970 (1.2) | 911 (1.1) | 765 (1.1) | 394 (0.9) |
| **Peak Stage 3, N (%)** | 2314 (0.6) | 498 (0.5) | 622 (0.7) | 532 (0.6) | 465 (0.6) | 197 (0.5) |

BMC: Bayview Medical Center; ED: Emergency Department; HCGH: Howard County General Hospital; JHH: Johns Hopkins Hospital; SH: Suburban Hospital; SMH: Sibley Memorial Hospital

**Table S2.** Predictor Variables Used

|  | **Total** | **Development** | **Validation** |
| --- | --- | --- | --- |
| **Total Visits** | 1124017 | 882746 | 241271 |
| **Age, C%** | 53.4 (20.0-91.0), 100.0% | 53.3 (20.0-91.0), 100.0% | 53.4 (20.0-91.0), 100.0% |
| **Age Subgroup, N (%)** |  |  |  |
| **18-44 years** | 424105 (37.7) | 333223 (37.7) | 90882 (37.7) |
| **45-64 years** | 340808 (30.3) | 267453 (30.3) | 73355 (30.4) |
| **65-74 years** | 153201 (13.6) | 120392 (13.6) | 32809 (13.6) |
| **>74 years** | 205903 (18.3) | 161678 (18.3) | 44225 (18.3) |
| **Sex at Birth, N (%)** |  |  |  |
| **Female** | 618866 (55.1), 100.0% | 485969 (55.1), 100.0% | 132897 (55.1), 100.0% |
| **Creatinine Baseline, mg/dL, C%** | 1.1 (0.6-1.7), 39.6% | 1.1 (0.6-1.7), 39.6% | 1.1 (0.6-1.7), 39.5% |
| **AKI initial presentation, C%** | 100.0% | 100.0% | 100.0% |
| **No AKI, N(%)** | 1028681 (91.5) | 807874 (91.5) | 220807 (91.5) |
| **Stage 1, N(%)** | 79867 (7.1) | 62763 (7.1) | 17104 (7.1) |
| **Stage 2, N(%)** | 15469 (1.4) | 12109 (1.4) | 3360 (1.4) |
| **Acute Kidney Injury**  **(Last 1 year), N(%)** | 32091 (2.9) | 25206 (2.9) | 6885 (2.9) |
| **Chief Complaint**  **(10 most frequent), C%** | 99.3% | 99.3% | 99.3% |
| **Abdominal Pain, N(%)** | 180260 (16.0) | 141527 (16.0) | 38733 (16.1) |
| **Chest Pain, N(%)** | 125492 (11.2) | 98497 (11.2) | 26995 (11.2) |
| **Shortness of Breath, N(%)** | 91677 (8.2) | 72038 (8.2) | 19639 (8.1) |
| **Blunt Trauma, N(%)** | 67674 (6.0) | 53169 (6.0) | 14505 (6.0) |
| **Psychiatric and Behavioral Diseases, N(%)** | 66047 (5.9) | 52097 (5.9) | 13950 (5.8) |
| **Musculoskeletal Non-Traumatic Disease, N(%)** | 52912 (4.7) | 41462 (4.7) | 11450 (4.7) |
| **Nausea, Vomiting, Diarrhea, N(%)** | 55244 (4.9) | 43536 (4.9) | 11708 (4.9) |
| **Genitourinary, N(%)** | 42057 (3.7) | 33010 (3.7) | 9047 (3.7) |
| **Weakness, N(%)** | 35646 (3.2) | 28067 (3.2) | 7579 (3.1) |
| **Substance Abuse, N(%)** | 32297 (2.9) | 25391 (2.9) | 6906 (2.9) |
| **Comorbidities, N (%)** | 371338 (33.0) | 291908 (33.1) | 79430 (32.9) |
| **Cerebrovascular Disease** | 57924 (5.2) | 45560 (5.2) | 12364 (5.1) |
| **Hemiplegia or Paraplegia** | 6200 (0.6) | 4846 (0.5) | 1354 (0.6) |
| **Mild Liver Disease** | 35873 (3.2) | 28165 (3.2) | 7708 (3.2) |
| **Moderate or Severe liver disease** | 4530 (0.4) | 3577 (0.4) | 953 (0.4) |
| **Diabetes without Complications** | 104566 (9.3) | 82138 (9.3) | 22428 (9.3) |
| **Diabetes with Complications** | 31137 (2.8) | 24575 (2.8) | 6562 (2.7) |
| **Renal Disease (mild or moderate)** | 52139 (4.6) | 41213 (4.7) | 10926 (4.5) |
| **Renal Disease (severe)** | 6124 (0.5) | 4878 (0.6) | 1246 (0.5) |
| **Cancer (any malignancy), Metastatic Solid Tumor** | 77435 (6.9) | 60821 (6.9) | 16614 (6.9) |
| **AIDS, HIV** | 35850 (3.2) | 28145 (3.2) | 7705 (3.2) |
| **Acute Myocardial Infarction** | 23673 (2.1) | 18640 (2.1) | 5033 (2.1) |
| **Chronic Pulmonary Disease** | 101860 (9.1) | 80312 (9.1) | 21548 (8.9) |
| **Congestive Heart Failure** | 65684 (5.8) | 51692 (5.9) | 13992 (5.8) |
| **Peripheral Vascular Disease** | 38009 (3.4) | 29929 (3.4) | 8080 (3.3) |
| **Peptic Ulcer Disease** | 6642 (0.6) | 5273 (0.6) | 1369 (0.6) |
| **Dementia** | 18464 (1.6) | 14470 (1.6) | 3994 (1.7) |
| **Rheumatoid Disease** | 14014 (1.2) | 10968 (1.2) | 3046 (1.3) |
| **Vital Signs, Mean (95% CI), C%** |  |  |  |
| **Temperature** | 98.1 (96.3-100.5), 97.0% | 98.1 (96.3-100.5), 97.0% | 98.1 (96.3-100.5), 97.1% |
| **Heart Rate** | 88.1 (56.0-131.0), 99.2% | 88.2 (56.0-131.0), 99.2% | 88.1 (56.0-130.0), 99.3% |
| **Respiratory Rate** | 17.9 (14.0-24.0), 98.2% | 17.9 (14.0-24.0), 98.2% | 17.8 (14.0-24.0), 98.2% |
| **Oxygen Saturation** | 97.6 (92.0-100.0), 98.7% | 97.6 (92.0-100.0), 98.7% | 97.5 (92.0-100.0), 98.8% |
| **Systolic Blood Pressure** | 136.8 (95.0-195.0), 99.8% | 136.8 (95.0-195.0), 99.8% | 136.8 (95.0-195.0), 99.8% |
| **Diastolic Blood Pressure** | 79.4 (52.0-112.0), 99.8% | 79.4 (52.0-112.0), 99.8% | 79.4 (52.0-112.0), 99.8% |
| **Labs, N Tested, Mean (95% CI), C%** |  |  |  |
| **Albumin, g/dL** | 1074767, 4.1 (2.8-5.1), 95.6% | 844111, 4.1 (2.8-5.1), 95.6% | 230656, 4.1 (2.8-5.1), 95.6% |
| **Anion Gap, mmol/L** | 1115200, 12.2 (5.0-21.0), 99.2% | 875799, 12.2 (5.0-21.0), 99.2% | 239401, 12.2 (5.0-21.0), 99.2% |
| **Blood Urea Nitrogen, mg/dL** | 1123543, 16.6 (6.0-45.0), 100.0% | 882369, 16.6 (6.0-45.0), 100.0% | 241174, 16.5 (6.0-45.0), 100.0% |
| **Creatinine, mg/dL** | 1123617, 1.0 (0.5-2.2), 100.0% | 882431, 1.0 (0.5-2.2), 100.0% | 241186, 1.0 (0.5-2.2), 100.0% |
| **Glucose, mg/dL** | 1123812, 126.0 (76.0-313.0), 100.0% | 882578, 126.0 (76.0-313.0), 100.0% | 241234, 125.9 (76.0-312.0), 100.0% |
| **hemoglobin, g/dL** | 1042133, 13.0 (8.3-16.6), 92.7% | 818573, 13.0 (8.3-16.6), 92.7% | 223560, 13.0 (8.3-16.6), 92.7% |
| **Potassium, mmol/L** | 1042426, 4.1 (3.2-5.2), 92.7% | 818575, 4.1 (3.2-5.2), 92.7% | 223851, 4.1 (3.2-5.2), 92.8% |
| **Lactate, mmol/L** | 168161, 2.1 (0.7-6.7), 15.0% | 131863, 2.1 (0.7-6.7), 14.9% | 36298, 2.1 (0.7-6.7), 15.0% |
| **Platelets, K/cu mm** | 1041287, 258.1 (115.0-465.0), 92.6% | 817894, 258.1 (115.0-465.0), 92.7% | 223393, 258.1 (115.0-465.0), 92.6% |
| **Sodium, mmol/L** | 1123783, 138.5 (130.0-145.0), 100.0% | 882563, 138.5 (130.0-145.0), 100.0% | 241220, 138.5 (130.0-145.0), 100.0% |
| **Specific Gravity** | 162937, 1.0 (0.0-1.0), 15.1% | 127987, 1.0 (0.0-1.0), 15.1% | 34950, 1.0 (0.0-1.0), 15.1% |
| **White Blood Cell Count, K/cu mm** | 1042130, 8.8 (3.7-18.4), 92.7% | 818568, 8.8 (3.7-18.4), 92.7% | 223562, 8.9 (3.7-18.5), 92.7% |
| C%: Indicator of predictor variable completeness, representing the percentage of encounters where at least one value was populated for the respective predictor variable prior to prediction timepoint. | | | |

**Table S3.** Sensitivity Analyses for validation-set AUC based on specific missingness model.

| **Predicted Outcome** | **Model Training Method** | **Missingness model (μ)** | | | | |
| --- | --- | --- | --- | --- | --- | --- |
|  |  | **-1** | **0** | **1** | **2** | **3** |
| AUC values for Any New or Progressive Acute Kidney Injury | Complete Case Training | 0.817 (0.807-0.827) | 0.844 (0.836-0.853) | 0.863 (0.857-0.870) | 0.874 (0.869-0.879) | 0.878 (0.874-0.883) |
|  | Missing Outcomes Assumed Negative | 0.804 (0.792-0.816) | 0.834 (0.825-0.844) | 0.862 (0.855-0.869) | 0.880 (0.875-0.885) | 0.888 (0.884-0.893) |
|  | Multiple Imputation | 0.816 (0.806-0.826) | 0.844 (0.835-0.852) | 0.863 (0.857-0.869) | 0.873 (0.868-0.879) | 0.878 (0.874-0.883) |
|  | Inverse Probability Weighting | 0.815 (0.804-0.827) | 0.842 (0.833-0.851) | 0.862 (0.856-0.869) | 0.874 (0.869-0.879) | 0.879 (0.874-0.883) |
| AUC values for Progression to Severe Acute Kidney Injury (Stage 2 or 3) | Complete Case Training | 0.829 (0.796-0.861) | 0.874 (0.852-0.896) | 0.903 (0.889-0.917) | 0.918 (0.908-0.928) | 0.924 (0.916-0.932) |
|  | Missing Outcomes Assumed Negative | 0.839 (0.807-0.869) | 0.880 (0.858-0.900) | 0.909 (0.895-0.921) | 0.924 (0.914-0.932) | 0.930 (0.922-0.937) |
|  | Multiple Imputation | 0.835 (0.803-0.865) | 0.878 (0.856-0.898) | 0.906 (0.892-0.918) | 0.920 (0.910-0.929) | 0.925 (0.917-0.933) |
|  | Inverse Probability Weighting | 0.835 (0.804-0.864) | 0.877 (0.855-0.897) | 0.905 (0.891-0.917) | 0.919 (0.909-0.928) | 0.924 (0.916-0.932) |

The missingness model assumes that the log likelihood of having the outcome observed is a function of underlying covariates plus a constant offset (μ, if the patient has AKI regardless of whether it is observed; zero, otherwise). Confidence intervals calculated using 10,000 bootstrapped replicates.

**Table S4.** Sensitivity Analyses for pairwise difference of validation-set AUC (relative to complete case training) based on specific missingness model.

| **Predicted Outcome** | **Model Training Method** | **Missingness model (μ)** | | | | |
| --- | --- | --- | --- | --- | --- | --- |
|  |  | **-1** | **0** | **1** | **2** | **3** |
| AUC difference for Any New or Progressive Acute Kidney Injury | Complete Case Training | - | - | - | - | - |
|  | Missing Outcomes Assumed Negative | -0.013 (-0.029,0.002) | -0.010 (-0.023,0.002) | -0.001 (-0.011,0.008) | 0.006 (-0.001,0.013) | 0.010 (0.003,0.016) |
|  | Multiple Imputation | -0.001 (-0.015,0.014) | -0.001 (-0.012,0.011) | -0.000 (-0.009,0.008) | -0.000 (-0.007,0.007) | -0.000 (-0.007,0.007) |
|  | Inverse Probability Weighting | -0.002 (-0.017,0.014) | -0.002 (-0.014,0.010) | -0.001 (-0.010,0.008) | -0.000 (-0.007,0.007) | 0.000 (-0.006,0.007) |
| AUC difference for Progression to Severe Acute Kidney Injury (Stage 2 or 3) | Complete Case Training | - | - | - | - | - |
|  | Missing Outcomes Assumed Negative | 0.010 (-0.035,0.054) | 0.006 (-0.025,0.036) | 0.005 (-0.014,0.024) | 0.006 (-0.008,0.019) | 0.006 (-0.005,0.017) |
|  | Multiple Imputation | 0.006 (-0.040,0.050) | 0.004 (-0.027,0.034) | 0.002 (-0.017,0.022) | 0.002 (-0.012,0.015) | 0.001 (-0.010,0.013) |
|  | Inverse Probability Weighting | 0.006 (-0.040,0.050) | 0.003 (-0.028,0.033) | 0.001 (-0.018,0.020) | 0.001 (-0.013,0.014) | 0.000 (-0.011,0.012) |

The missingness model assumes that the log likelihood of having the outcome observed is a function of underlying covariates plus a constant offset (μ, if the patient has AKI regardless of whether it is observed; zero, otherwise). Confidence intervals calculated using 10,000 bootstrapped replicates.

**Table S5:** Model Performance (AUC) by ED Study Site, Sex, Race, and Ethnicity

| **Predicted**  **Outcome** | **Stratification** | **Population** | **Complete Case Training** | **Missing Outcomes**  **Assumed Negative** | **Multiple Imputation** | **Inverse Probability**  **Weighting** |
| --- | --- | --- | --- | --- | --- | --- |
| **Any New or Progressive Acute Kidney Injury** | **Validation Cohort** | 81231 | 0.82(0.81-0.82) | 0.80(0.80-0.81) | 0.82(0.81-0.82) | 0.81(0.81-0.82) |
|  | **Hospital** |  |  |  |  |  |
|  | JHH | 20664 (25.0%) | 0.8(0.79-0.82) | 0.78(0.77-0.8) | 0.8(0.79-0.81) | 0.79(0.78-0.81) |
|  | BMC | 15342 (19.0%) | 0.8(0.79-0.82) | 0.79(0.78-0.81) | 0.81(0.79-0.82) | 0.8(0.79-0.82) |
|  | HCGH | 17361 (21.0%) | 0.82(0.81-0.83) | 0.81(0.79-0.82) | 0.82(0.81-0.83) | 0.82(0.81-0.83) |
|  | SH | 17517 (22.0%) | 0.83(0.81-0.84) | 0.83(0.81-0.84) | 0.83(0.82-0.84) | 0.83(0.81-0.84) |
|  | SMH | 10347 (13.0%) | 0.84(0.82-0.85) | 0.82(0.81-0.84) | 0.83(0.82-0.85) | 0.84(0.82-0.85) |
|  | **Sex** |  |  |  |  |  |
|  | Male | 39343 (48.0%) | 0.81(0.81-0.82) | 0.8(0.79-0.81) | 0.81(0.8-0.82) | 0.81(0.8-0.82) |
|  | Female | 41888 (52.0%) | 0.82(0.81-0.83) | 0.81(0.8-0.82) | 0.82(0.81-0.83) | 0.82(0.81-0.82) |
|  | **Race** |  |  |  |  |  |
|  | White | 43567 (54.0%) | 0.82(0.81-0.82) | 0.8(0.8-0.81) | 0.81(0.81-0.82) | 0.81(0.8-0.82) |
|  | Black | 25838 (32.0%) | 0.81(0.8-0.82) | 0.79(0.78-0.8) | 0.81(0.8-0.82) | 0.8(0.79-0.81) |
|  | Asian | 2963 (4.0%) | 0.83(0.79-0.86) | 0.82(0.79-0.86) | 0.83(0.8-0.87) | 0.82(0.79-0.85) |
|  | Other | 8863 (11.0%) | 0.84(0.82-0.86) | 0.84(0.82-0.86) | 0.84(0.82-0.86) | 0.84(0.82-0.86) |
|  | **Ethnicity** |  |  |  |  |  |
|  | Hispanic | 4128 (5.0%) | 0.85(0.82-0.88) | 0.83(0.8-0.86) | 0.85(0.82-0.88) | 0.86(0.83-0.89) |
|  | Not Hispanic | 77103 (95.0%) | 0.82(0.81-0.82) | 0.8(0.8-0.81) | 0.81(0.81-0.82) | 0.81(0.8-0.82) |
| **Progression to Severe Acute Kidney Injury (Stage 2 or 3)** | **Validation Cohort** | 81231 | 0.87(0.86-0.88) | 0.87(0.86-0.88) | 0.87(0.86-0.89) | 0.87(0.86-0.88) |
|  | **Hospital** |  |  |  |  |  |
|  | JHH | 20664 (25.0%) | 0.86(0.84-0.88) | 0.86(0.83-0.88) | 0.86(0.84-0.89) | 0.86(0.84-0.89) |
|  | BMC | 15342 (19.0%) | 0.88(0.86-0.9) | 0.87(0.85-0.89) | 0.88(0.86-0.9) | 0.88(0.86-0.9) |
|  | HCGH | 17361 (21.0%) | 0.86(0.84-0.89) | 0.86(0.84-0.88) | 0.86(0.84-0.88) | 0.86(0.83-0.88) |
|  | SH | 17517 (22.0%) | 0.88(0.85-0.9) | 0.88(0.85-0.9) | 0.88(0.86-0.9) | 0.87(0.85-0.9) |
|  | SMH | 10347 (13.0%) | 0.91(0.88-0.93) | 0.9(0.88-0.93) | 0.9(0.88-0.93) | 0.9(0.88-0.93) |
|  | **Sex** |  |  |  |  |  |
|  | Male | 39343 (48.0%) | 0.88(0.86-0.89) | 0.87(0.86-0.89) | 0.88(0.86-0.89) | 0.88(0.86-0.89) |
|  | Female | 41888 (52.0%) | 0.87(0.86-0.89) | 0.87(0.85-0.88) | 0.87(0.86-0.89) | 0.87(0.85-0.88) |
|  | **Race** |  |  |  |  |  |
|  | White | 43567 (54.0%) | 0.88(0.86-0.89) | 0.87(0.86-0.89) | 0.88(0.86-0.89) | 0.87(0.86-0.89) |
|  | Black | 25838 (32.0%) | 0.87(0.85-0.88) | 0.86(0.85-0.88) | 0.87(0.85-0.88) | 0.86(0.85-0.88) |
|  | Asian | 2963 (4.0%) | 0.89(0.86-0.93) | 0.89(0.86-0.92) | 0.9(0.86-0.93) | 0.9(0.87-0.93) |
|  | Other | 8863 (11.0%) | 0.88(0.84-0.91) | 0.88(0.85-0.91) | 0.88(0.85-0.91) | 0.88(0.84-0.91) |
|  | **Ethnicity** |  |  |  |  |  |
|  | Hispanic | 4128 (5.0%) | 0.9(0.84-0.95) | 0.9(0.85-0.95) | 0.9(0.85-0.95) | 0.9(0.85-0.95) |
|  | Not Hispanic | 77103 (95.0%) | 0.87(0.86-0.88) | 0.87(0.86-0.88) | 0.87(0.86-0.88) | 0.87(0.86-0.88) |
